# Analyzing human factors affecting severe maternal morbidity (SMM) using fuzzy Bayesian network (FBN)

**DOI:** 10.3389/fmed.2025.1566625

**Published:** 2026-01-02

**Authors:** Maryam Feiz-Arefi, Fereydoon Laal, Amin Babaei-Pouya, Homeyra Mohammadi Darmiyan, Zahra Pajohideh, Akram Ajam

**Affiliations:** 1 Department of Occupational Health Engineering, School of Health, Social Determinants of Health Research Center, Gonabad University of Medical Sciences, Gonabad, Iran; 2 Department of Occupational Health and Safety Engineering, School of Health, Social Determinants of Health Research Center, Birjand University of Medical Sciences, Birjand, Iran; 3 Department of Occupational Health Engineering, School of Health, Ardabil University of Medical Sciences, Ardabil, Iran; 4 Department of Occupational Health and Safety Engineering, Birjand University of Medical Sciences, Birjand, Iran; 5 Department of Midwifery, Shoushtar Faculty of Medical Sciences, Shoushtar, Iran; 6 Gonabad University of Medical Sciences, Gonabad, Iran

**Keywords:** severe maternal morbidity (SMM), fuzzy Bayesian network (FBN), hospital, care, health center

## Abstract

**Background and aims:**

Severe maternal morbidity (SMM) is one of the key indicators for assessing the quality of obstetric care and is frequency associated with human error. This study aimed to analyze the human factors contributing to SMM using fault tree analysis (FTA) and fuzzy Bayesian network (FBN).

**Methods:**

The present study was conducted using morbidity file data obtained from Birjand and Gonabad universities supplemented with expert interviews. First, basic events were identified and the fault tree structure was validated. Error probabilities were estimated using three approaches: Fast Fourier Transform (FFT), and FBN with and without Common cause failures (CCFs). The L-NOR gate was used to reduce the complexity of conditional probability tables (CPT) and to capture dependencies among factors. Sensitivity analysis and the strength of influence of contributing factors were assessed.

**Results:**

The main contributors to SMM were the delay in initiating emergency resuscitation efforts, inadequate management of obstetric hemorrhage, and poor team coordination, which showed the highest strength of influence on SMM occurrence. The final SMM probability was 0.0196 in FFT, 0.0193 in FBN without CCFs, and 0.0167 in FBN with CCFs.

**Conclusion:**

Integrating FTA and FBN methods, particularly with the L-NOR gate, overcomes limitations of traditional approaches and enables more accurate modeling of cause-and- effect relationships in complex systems. Strengthening team coordination, appropriate management of hemorrhage, and implementation and enforcement of standard protocols are among the suggested strategies to reduce SMM. These findings provide valuable insights for policy-making and strategies to improve obstetric care.

## 1 Introduction

Severe maternal morbidity (SMM) is defined as “unintended consequences of the birth process that result in significant short- or long-term consequences for women’s health” and is 50–100 time more common than maternal mortality (1, 2). There are approximately 70 SMM cases for every pregnancy-related death. More than 50,000 women were affected by SMM in 2014 (3). Internationally, SMM and near-fatality surveys are being used as a new indicator to assess the quality of maternal care and as an alternative strategy to reduce maternal morbidity (4). Maternal morbidity is a more useful indicator of maternal care than mortality (5).

Severe maternal complications may occur during pregnancy, labor, or postpartum due to hypertensive disorders, hemorrhage, infection, and other serious or life-threatening conditions affecting the woman or the infant. Human error is recognized as a direct cause of maternal death and morbidity (6). Human errors occur when human actions deviate from accepted norms, limits, and standards, thereby compromising patient safety and system efficiency (7). Healthcare and hospital environments are prone to human error due to high workload, fatigue, multitasking, and complex information processing and decision-making (8, 9). Medical error has been reported as the third leading cause of death in the United States (10). Medical errors encompass diagnostic errors, medication errors, nursing care errors, surgical errors, and skill deficiencies (11). Human factors have been implicated as key contributors to maternal deaths worldwide (12). According to statistics provided by the Director General of the Health and Population Office of the Ministry of Health of Iran, 60% of maternal deaths (1999–2005) were due to human errors, including medical, obstetric, and nursing errors (13). With the increase in the incidence of medical errors, many efforts have been made to eliminate or reduce them since the late 1990s. Human errors have been the focus of safety studies since 1950. While identifying maternal risk factors is a critical step toward preventing adverse outcomes, understanding how human errors contribute to these outcomes is equally important. Techniques for predicting human error were developed in 1964 (11). There are various methods for risk assessment and root cause ranking of events, among the most important are fault tree analysis (FTA) approaches and Bayesian networks (BN). The application of systematic techniques such as FTA and BNs enables researchers to model the relationships between human errors, contextual factors, and clinical outcomes, and to quantitatively estimate their likelihood of occurrence.

The FTA technique, as one of the most widely used and popular risk assessment techniques, also has some weaknesses. Among these weaknesses are its limitation to binary variables, static and inflexible structure, and the use of deterministic logic gates. The BN approach has received increasing attention in recent years as it overcomes these limitations. BN are belief-based networks that represent cause-and-effect relationships between multiple variables (14). The purpose of designing, building, and analyzing BN is to make decisions under uncertain and highly uncertain conditions. Each BN consists of two qualitative and quantitative parts. The qualitative part includes variables and the cause-and-effect relationships between them in the form of directed arcs. Their quantitative part includes conditional probability tables (CPTs) defined for each variable. BN have been used in accident analysis (15), human reliability assessment (16), hospital risk management, and patient safety (17).

Bayesian network has been used in numerous studies in healthcare to analyze and predict neonatal complications (18), predict out-of-hospital cardiac arrest (19), manage older adult care, and predict hospitalization period (20). Prior studies of maternal morbidity using BN have aimed to understand the joint effects of clinical, demographic, and regional factors on SMM (21). The role of human factors in the occurrence and prevention of these factors has not been studied. Rather most research and review articles in Iran and other countries have been conducted with the aim of identifying the clinical causes of maternal morbidity and morbidity (22–26) with comparatively few examining the contribution of human or organizational errors to maternal and fetal morbidity (27). Of the available studies, only a few have specifically modeled human factors in SMM using quantitative risk modeling approaches. Therefore, the present study aims to analyze the human factors contributing to SMM using the fault tree analysis (FTA) and a fuzzy Bayesian network (FBN) approach. This combined method enables the identification and quantification of human errors within complex systems, offering a more comprehensive understanding of their role in SMM and potential strategies for prevention.

## 2 Materials and methods

**FIGURE 1:**
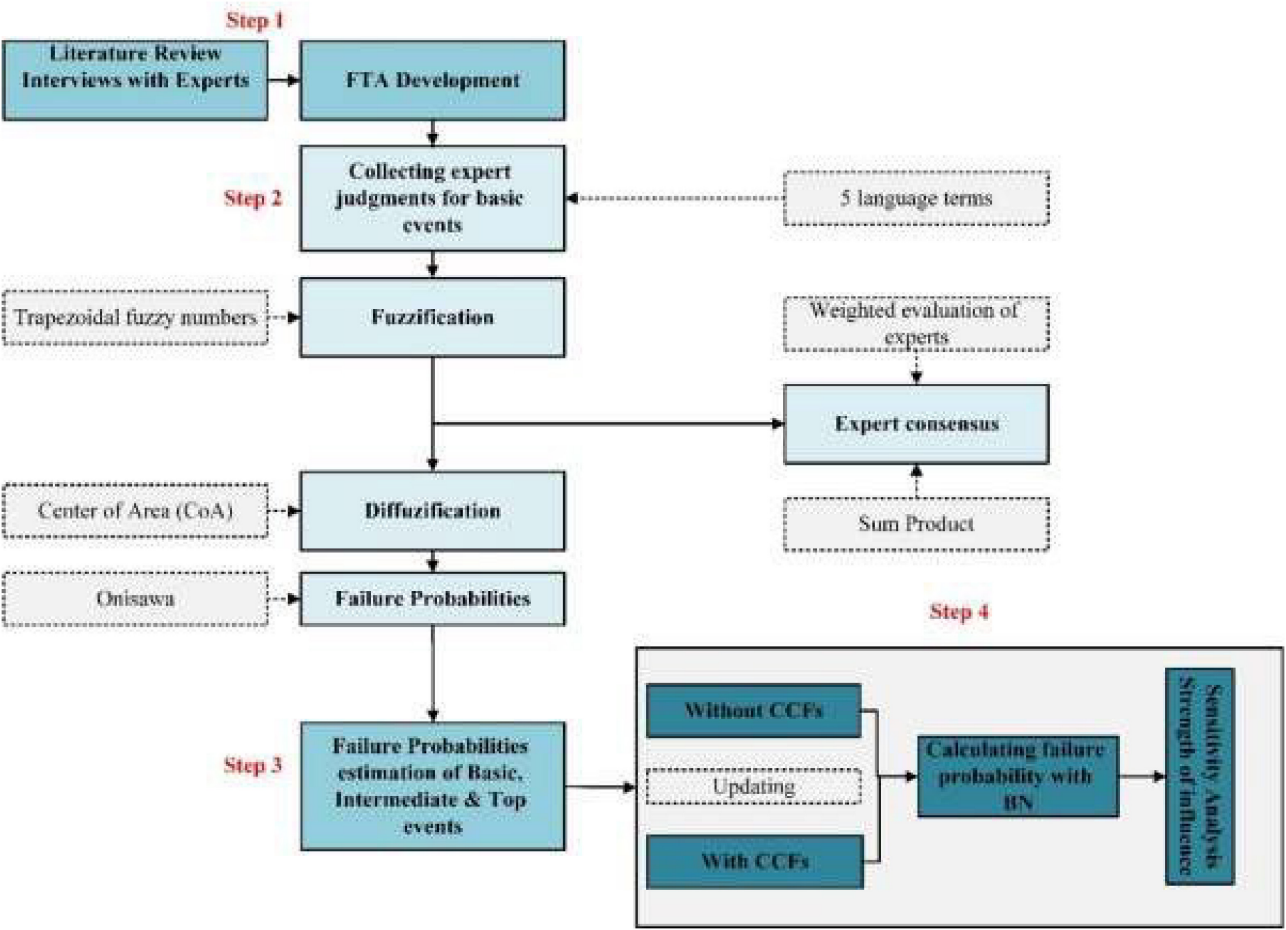
Study flowchart.

Figure 1 shows the overall flowchart of the study. The implementation of this study consisted of 4 steps.

**Step1. Development of the Fault Tree Analysis (FTA)**

Fault Tree Analysis identifies the main roots by dividing faults into sub-branches to make it easier to improve efficiency and reduce faults. Coordination was made with the relevant units to implement this study (28). The files and documents of the Morbidity Committee of the Vice- Chancellor of Medicine of the Universities (Gonabad and Birjand) over the last 10 years, along with similar studies, were reviewed to identify human errors contributing to SMM. Using these data, a Fault Tree diagram was constructed to represent the events leading to SMM.

Step2. Fuzzy logic analysis for estimating the probability of basic events

2a: Expert elicitation and Weighting of expert input

Due to the lack of empirical data on basic event probabilities, expert judgment was used. In this step, a structured questionnaire was designed based on identified fault tree root causes. The questionnaire was distributed to two expert panels in Gonabad and Birjand (total *n* = 18; approximately nine per city) (Table 1). The experts’ opinions were collected as verbal variables with a 5-point Likert scale with trapezoidal fuzzy numbers (very low, low, medium, high, and very high).

**TABLE 1:** Weight factor of the study experts.

Row	Educational level	Organizational position	Work experience (y)	Experts’ weight
1	PhD	Faculty member	21	0.0769
2	Specialist	Obstetrics and gynecology surgeon	12	0.0833
4	Bachelor	Expert at the health deputy headquarters	17	0.0448
13	Bachelor	Morbidity expert of deputy of treatment	16	0.0448
17	Bachelor	Health center midwife	15	0.0448
18	Bachelor	Hospital midwife	16	0.0448

Expert responses were weighted based on professional experience, age, organizational role, and educational background, using the Laal et al.’s study (29). For more information, see Table 2 of Laal’s study (29). These weights were applied in the probabilistic analysis to enhance the accuracy and validity of the results.

**TABLE 2:** Failure probability of maternal morbidity basic events with fuzzy logic.

Basic event		Descriptions	Error probability
Lowest of error probability	X2	Failure to train to control high-risk situations	0.00023
	X3	Failure to follow up with high-risk mothers	0.00031
	X6	Incomplete registration of information	0.00028
	X13	Failure to check the necessary documents in the file (examination, operating permit, etc.)	0.00035
	X15	Failure to monitor the pregnant mother’s condition	0.00037
Highest of error probability	X16	Failure to control bleeding	0.00073
	X19	Failure to announce the Cardiopulmonary resuscitation (CPR) code on time	0.00074
	X20	Failure in interdepartmental coordination to perform CPR	0.00074
	X24	Failure to control bleeding	0.00073
	X34	Failure to check for bleeding	0.00073

The experts were specialists in the field of maternal morbidity, including health center staff, hospital obstetricians and gynecologists, and experts of the morbidity committee of the deputy of treatment.

Each had acceptable knowledge in the field of care of pregnant mothers, possible errors in providing care, and the probability of occurrence of these errors. The experts were familiar with the fault tree structure or were given explanations about the main approach of this study after entering the study. In this step, some basic events or root causes were added or deleted. The fault tree was validated with the opinions of experts. Fuzzy logic was used to determine the probability of basic events.

Accordingly, the present study utilized this hybrid approach for both expert consensus and defuzzification, as detailed in Equation 1.


$$Z_{i}=g_{x}=\sum_{j=1}^{n}w_{j}\,f_{i\,j}\,i=1,2,\ldots ,n\,j=1,2,\ldots ,m$$ (1)

Where Z_*i*_ is the consensus fuzzy number for BEs, w_*j*_ is Expert Weight j, and f_*ij*_ is Fuzzy numbers of the BE i according to the expert j.

2b: Defuzzification and conversion to probabilities

Various techniques are available to consolidate expert judgments, such as the linear opinion pool, the max–min Delphi method, and the sum–product approach (30–32). In this study, we employed the sum–product approach. Previous research by Yazdi and Zarei, which focused on managing uncertainty in safety risk assessments and evaluating multiple aggregation strategies, demonstrated that the Center of Area (CoA)/Sum-product approach provided superior performance in terms of computational efficiency, reliability, and processing time (33).

In the next step, the obtained fuzzy numbers were defuzzified. There are various methods for defuzzifying fuzzy numbers. Defuzzification of fuzzy numbers is an important method for decision making in fuzzy environment. In this study, the CoA method was used for defuzzification proposed by Sugeno (34).

Equations 2, 3 represents the CoA method for defuzzifying fuzzy numbers. The values obtained from the previous step are called failure possibility. Using the Onisawa equations (Equations 4, 5), the failure possibility was converted to failure probability (35).


$$X^{*}=\frac{\int \mu_{i}\,\left(x\right)\,x\,dx}{\int \mu_{i}\,\left(x\right)\,dx}$$ (2)

In this relation, X* or CFP is the defuzzy output, μ_*i*_(*x*) is the aggregated membership function, and x is the output variable. To defuzzify trapezoidal fuzzy numbers based on the CoA, the summarized Equation 3 is also used.


X=*13(a4+a3)2-a4⁢a3-(a1+a2)2+a1⁢a2(a4+a3-a1-a2) (3)

$$F\,P=\left\{\begin{array}{c} \frac{1}{10^{k}},C\,F\,P\neq 0\\ 0,C\,F\,P=0 \end{array}\right\}$$ (4)

$$\text{K}=\left[\left(\frac{1-P\,s\,1/3}{P\,s}\right)\right]\times 2.301$$ (5)

In these relations, FP: probability rate of each basic event, CFP or X*: Crisp Failure possibility resulting from the defuzzification step, K: an intermediate variable that is a function of CFP and Trapezoidal fuzzy numbers: ´*A* = *a*_1_, *a*_2_, *a*_3_, *a*_4_

Step3. Calculating the Probability of Intermediate and Main Events with Fuzzy Fault

**Tree (FFT)**

Fault tree analysis is one of the most important and widely used methods in risk and system safety assessment, which can be used to determine the most important root causes of an adverse incident or event. In this method, various types of logic gates, including “AND” and “OR,” were used to establish relationships between basic, intermediate, and main events. For quantitative analysis, the rules of Boolean algebra were used in this method. The ease of calculations and understandable graphics of this method have made it one of the popular techniques among safety and system engineers. However, this method also has limitations. For example, in this method, all variables are binary or two-state, and the gates used in this method are deterministic. On the other hand, the type of analysis and belief expansion in this method is one-way, from basic events to the main event (36). To cover these weaknesses, BNs will be used in this study.

Step4. Probability modeling with BN and sensitivity analysis

4a: Construction of the Bayesian Network

A BN is composed of nodes, directed edges, and CPTs, which collectively represent random variables and their conditional dependencies. Its capability to revise prior probabilities and incorporate common cause failures (CCFs), within a system makes the BN framework particularly effective for risk analysis (37). The CPT in a BN represents the probability of each state of a node given the combination of states of its parent nodes. These tables are typically populated using historical data (when available) or expert judgment through structured methods such as Delphi (38). In software tools such as GeNIe. In this study, based on the validated structures of the fault tree, it was supplemented with expert opinions and gate type to indicate the type and intensity of the dependency between nodes.

In this study, after estimating probabilities using fuzzy logic, the BN was used due to its suitable graphical representation and strong reasoning power. This approach has been used as a reliable methodology in various studies (39–41). In the present study, basic, intermediate, and main events were considered as root nodes, intermediate nodes, and central nodes, respectively, and then CPTs were created for different nodes (42).

4b: Updating and probability inference

Based on the conditional relationships between variables and the application of chain rules, Jensen and Nielsen (43) defined a BN as a probabilistic distribution over a set of variables, as formulated in Equation 6.


$$P\,\left(U\right)=\prod_{i=1}^{n}P\,\left(X_{i}|P_{a}\,\left(X_{i}\right)\right)$$ (6)

In Equation 6, Pa(Xi) is the parent set of the variable Xi and P(U) is the joint probability distribution of the variables.

The BN is updated using a new set of evidence (E) of initial probabilities according to Equation 7 (29).


$$P\left(U|E\right)=\frac{P\left(U\right).P\left(E|U\right)}{\Sigma UP\left(U\right).P\left(E|U\right)}=\frac{P\,\left(U,E\right)}{\Sigma \,U\,P\,\left(U,E\right)}$$ (7)

These formulas (Equations 6, 7) represent Bayes’ theorem, where prior probabilities are updated with new evidence to yield posterior probabilities.

In this study, NoisyMax nodes were also used to estimate the probability of events with CCFs, which allow them to specify their interactions with their parents simply and require fewer parameters. NoisyMax allows us to model situations where several independent factors contribute to the same outcome, such as multiple simultaneous clinical errors leading to morbidity.

4c: Sensitivity analysis and identification of critical events.

Sensitivity analysis can be used to identify the most important root nodes that cause system failure because the importance of root nodes in the BN varies (29). Also, the strength of influence and sensitivity analysis was performed for different events in two cases (without CCFs and with CCFs). The influence strength is calculated from the CPT of the child node. Events with higher influence strength were considered critical contributors to maternal morbidity.

The values are entered manually, and the software performs normalization and facilitates result analysis. Essentially it expresses the distance between different conditional probability distributions on the child node conditional on the states of the parent node.

## 3 Results

**FIGURE 2:**
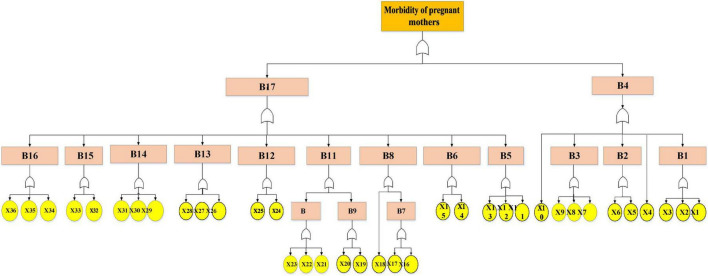
Fault tree analysis (FTA) of maternal morbidity.

The present study analyzes the human causes affecting severe morbidity in pregnant mothers using the FTA technique and fuzzy Bayesian network (FBN). Accordingly, the results of the analysis and findings of the study are reported as follows. According to Table 1, in this study, 18 experts expressed their opinions on the basic events that were finalized in Figure 2. The results are summarized here. Experts 2 and 3 were obstetric and gynecological surgeons with the highest weight (0.0833). The lowest weight (0.0448) were related to expert at the health deputy headquarters, morbidity expert of deputy of treatment, health center midwife and hospital midwife. According to the study method, it can be said that experts with higher degrees, age, and education were assigned higher weight. This can highlight the importance of the opinions of these experts in later stages. After validating the fault tree in the occurrence of maternal morbidity, the number of identified basic events was 36 and the intermediate events were 17 (Figure 2).

The various events are summarized in Table 2, and some of these events are described below. Delayed activation of the CPR code, systemic delays in emergency response, lack of interdepartmental coordination, and inadequate control of hemorrhage are among the most critical patient safety concerns. Late initiation of maternal resuscitation or slow response to urgent conditions such as severe bleeding or fetal bradycardia can lead to life-threatening complications, including coma or death. Moreover, insufficient communication and coordination between obstetrics, anesthesia, and resuscitation teams–caused by the absence of clear protocols and predefined pathways–further reduce the efficiency of emergency management. In addition, failure to prioritize and adequately control maternal hemorrhage before proceeding with other interventions significantly increases the risk of adverse outcomes.

The probability of occurrence of basic events was calculated with FFT. According to Table 2, the highest probability of basic events based on FFT was related to X19 (0.00074), and the lowest was to X2 (0.00023). Here are 5 events with high and low probability. More details of Table 2, like the previous table, are provided in the Supplementary Materials section. The probability of occurrence of intermediate events based on FFT is also seen in the second column of Table 3.

**TABLE 3:** Probabilities of intermediate events and main events with FFT and FBN (without/with CCFs).

Intermediate event	“FFT”	FBN without CCFs	FBN with CCFs
Error in providing pre-pregnancy services (B1)	0.000955703	0.000955702	0.000955702
Admission and record creation (B2)	0.000775860	0.000775859	0.000775859
Error in examination of pregnant mother (B3)	0.001719022	0.001719022	0.000531952
Error in health center care (B4)	0.004562705	0.004562705	0.003378541
Admission and record creation (B5)	0.001473291	0.001473290	0.001473290
Error in monitoring the symptoms of a pregnant mother (B6)	0.000756857	0.000756856	0.000756856
Lack of risk control (B7)	0.001268608	0.001268607	6.07061E-05
Error in managing critical cases (B8)	0.001798934	0.001798934	0.000591673
Failure to perform CPR (B9)	0.001975698	0.001480451	0.001790037
Unsuccessful CPR (B10)	0.003771078	0.001975697	0.001975697
Error in resuscitation procedures (B11)	0.000955703	0.003453224	0.003761757
Error in managing postpartum conditions (B12)	0.001175676	0.001175676	0.001175676
Error in managing emergency situations (B13)	0.001800938	0.001800937	0.001238291
Error in anesthesia procedures (B14)	0.001148560	0.001148560	0.001148560
Error in cesarean procedures (B15)	0.001283588	0.001283588	0.001357399
Error in monitoring the mother’s condition postpartum (B16)	0.002003674	0.002003674	0.002003674
Error in hospital (B17)	0.015113160	0.014798925	0.013426428
Top event	0.019606908	0.019294107	0.016759608

After calculating the failure probabilities of the basic events, the corresponding events were transferred to the BN according to the working method. The BN analysis results are also presented separately, with and without CCFs.

### 3.1 Analysis of BN results without common cause failures (CCFs)

**FIGURE 3:**
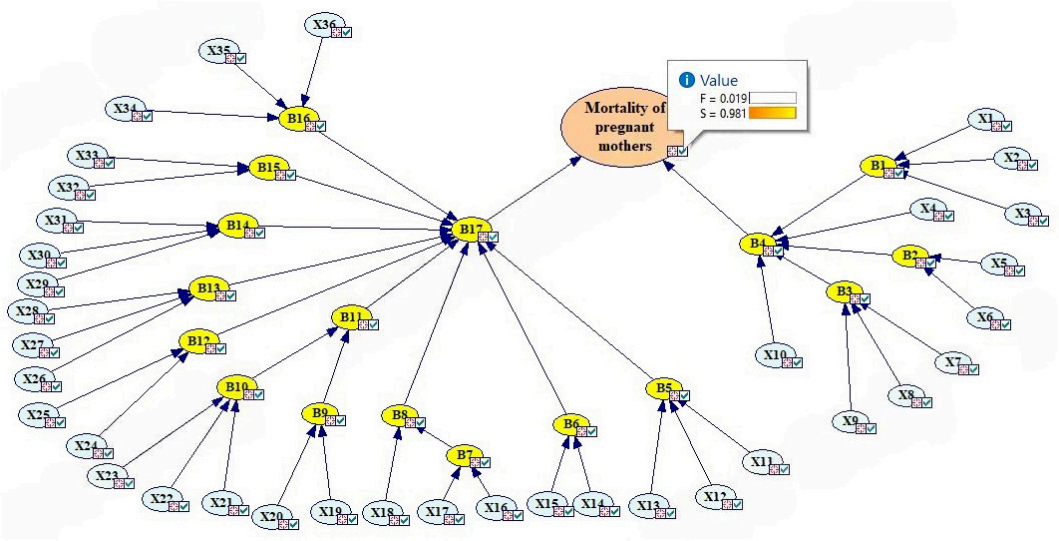
Updating the probabilities of maternal morbidity with the BN. Created using GeNIe Modeler, BayesFusion, LL (http://www.bayesfusion.com/), licensed under Academic Software License.

First, the analysis was performed without CCFs. According to Figure 3, the failure probability of the final event that leads to maternal morbidity was estimated to be 0.019294107 (column 3 of Table 3). Figure 3 shows the general structure of the FBN. In this figure, the basic events (blue) are linked to the intermediate events (yellow) according to the method. The probabilities of the basic events are the same as the fuzzy logic probability. The probabilities of the intermediate events were obtained according to Table 3. According to Table 3, the highest failure probabilities without CCFs were related to B17 (error in hospital care) and B4 (error in health center care), respectively. Of the intermediate events, lower levels of B11 (error in resuscitation) and B16 (error in postpartum maternal monitoring status) also had high probabilities. Also, event B3 (error in examination of pregnant mother) was the most important event related to B4 (error in health center care).

**FIGURE 4:**
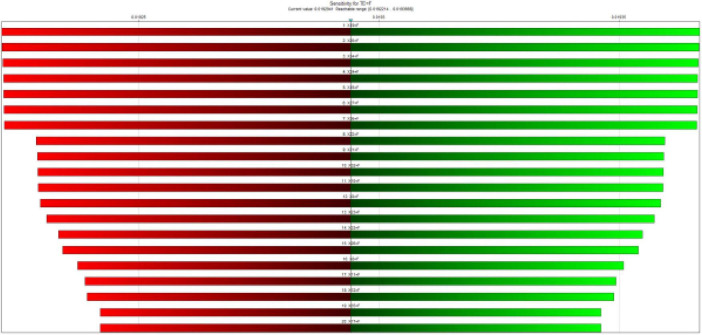
Sensitivity analysis to identify the most important critical events.

The results of the sensitivity analysis showed that the most critical baseline events in the morbidity of pregnant mothers were X19 (Failure to announce the CPR code on time), X20 (Failure in interdepartmental coordination to perform CPR), X34 (Failure to check for bleeding), X24 (Failure to control bleeding), X16 (Failure to control bleeding), and X27, respectively (Figure 4).

The bar color indicates the direction of change in the target state, red indicates a negative change and green indicates a positive change.

**FIGURE 5:**
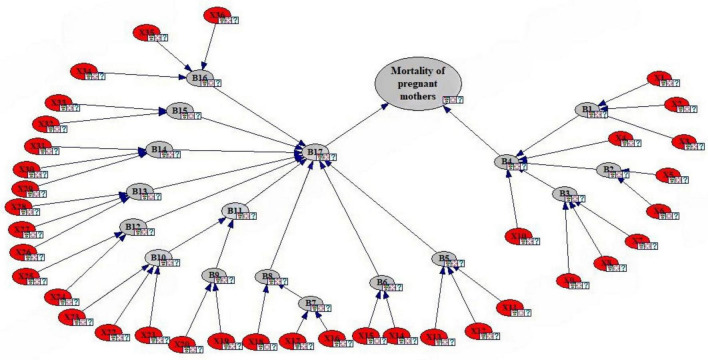
Strength of influence of different events. Created using GeNIe Modeler, BayesFusion, LL (http://www.bayesfusion.com/), licensed under Academic Software License.

The strength of influence of different events shows that the results were almost in line with the sensitivity analysis, but the influence strength of events X34 and X27 was lower than the sensitivity analysis (Figure 5). X32, X33, X5, X6, X14, X15, X16, X17, X18, X19 and X20 also had influence strength of approximately 0.5. Failure to special care for critically ill patients (X36), Failure of experts to perform CPR (X26), and the inability of staff to perform CPR (X21) were also important events in the sensitivity analysis. The arch has different thicknesses, depending on the strength of the penetration between the nodes that connect them. The influence strength is calculated from the CPT of the child node.

This is due to the difference in how each method quantifies the importance of the node, and using the two methods can identify the most sensitive events with different approaches. Therefore, the existing difference is due to methodological divergence and is not considered a flaw.

### 3.2 Analysis of BN results with common cause failures (CCFs)

Multiple events that influence each other and the estimation of these effects in the study were also examined. For example, event X5 can influence X8, whose conditional probability of failure was estimated by fuzzy logic and expert opinions to be 2.37E-05. The final probabilities were calculated using these values and specifying the interactions between events (Table 4).

**TABLE 4:** Conditional probabilities of events with CCFs.

Row	Common cause failures (CCFs)	Conditional probabilities of CCFs
1	X5 → X9	2.68E-05
2	X5 → X8	2.37E-05
3	X11, X12, X13 → X16	2.94E-05
4	X11, X12, X13 → X17	2.98E-05
5	X11, X12, X13 → X27	1.72E-04
6	X21, X26 → X19	1.05E-03
7	X22, X23 → X33	6.93E-04

**FIGURE 6:**
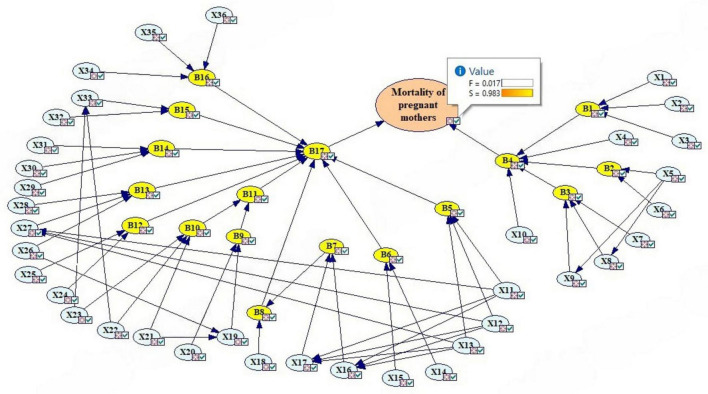
Updating maternal morbidity probabilities with a BN with CCFs. Created using GeNIe Modeler, BayesFusion, LL (http://www.bayesfusion.com/), licensed under Academic Software License.

As can be seen in Figure 6, the final probability was obtained with CCFs of 0.016759608 (second case), which is less than the first case. Column 4 of Table 3 shows the probabilities of events with CCFs, where the intermediate events that have been influential in this process have different probabilities than the previous case, while the probabilities of events such as B1 and B6 have not changed. Modeling CCFs has reduced the overall estimation probability, which could be due to the gate type in FFT and differences in CPT completion in FBN.

**FIGURE 7:**
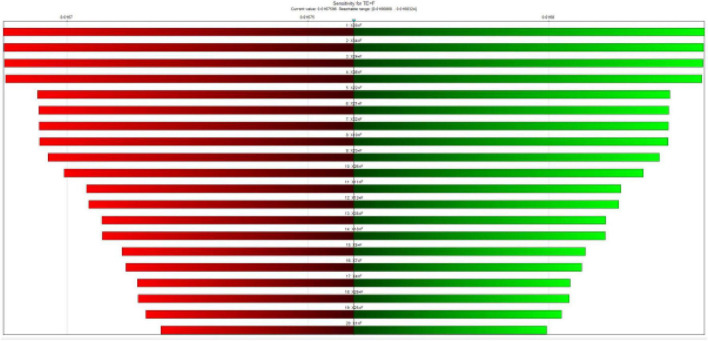
Sensitivity analysis to identify the most important critical events with CCFs.

In this case, sensitivity analysis was also performed. The results showed that events X20, X34, X24, X36, X22 and X21 were the most important events of the study (Figure 7). X28 and X29 were the least important events of the study. Meanwhile, the strength of influence of the various events was almost the same as in the previous case.

## 4 Discussion

The present study analyzed human factors contributing to SMM using a combined FTA and FBN approach. The results demonstrate that human factors, including diagnostic errors, poor interdepartmental coordination, and poor hemorrhage control, are the most important root causes of SMM. These findings indicate the importance of investigating and managing human errors in maternity care settings as a central component of improving maternal safety. Our findings are consistent with previous studies finding that human errors in clinical settings are among the most important causes of maternal morbidity (44).

The results showed that the probability of B17 (hospital care errors) is higher than B4 (health center care errors). The higher rate of care errors in hospitals compared to primary health centers can be explained by their different approaches. Health centers focus on prevention, screening, and early detection of risks, allowing more time for decision-making and reducing errors. In contrast, hospitals deal with high-risk patients and critical situations, performing complex and urgent interventions under time pressure and multidisciplinary coordination, which increases the likelihood of errors. This does not necessarily indicate lower quality but rather reflects the greater complexity of hospital care. Strengthening preventive interventions can reduce hospital workload and ultimately decrease errors at the health system level. The results of this study point to error in examination of pregnant mother (B3) as a key factor.

Inadequate prenatal screening or misdiagnosis of complications can lead to irreversible consequences. However, it is practical for doctors and policymakers to pay attention to this and provide preventive measures.

Several studies consistently identify hemorrhage as the leading contributor to SMM, which our BN model also confirms (26, 45–47). Geller et al. found that bleeding and hypertensive disorders were the most important conditions contributing to SMM in all regions (46). Based on the results of Geller et al. factors such as failure to identify high-risk situations, delay in announcing the CPR code, and failure to control bleeding were among the most critical factors affecting the occurrence of SMM (46).

Other direct causes of maternal death in Tajvar et al. study included hypertension, infection, abortion and ectopic pregnancy, labor obstruction, and uterine rupture (47). Zalvand et al. conducted a review study to investigate the causes of maternal morbidity between 2003 and 2017 in Iran. Bleeding, hypertensive disorders, and circulatory diseases were the main causes of morbidity. The most important determinants of maternal morbidity in Iran were stated as gravity, type of delivery, socio-economic status of mothers, place of birth, death, and postpartum care (26). Structured rapid response protocols, team training for bleeding management, and interdepartmental maneuvers are recommended.

In this study, the combination of FTA and FBN methods provided a unique and efficient framework for identifying and analyzing the root causes of SMM. This method was able to identify not only the contributing factors, but also reveal the complex interactions between factors.

In the present study, given that there was no database for root events identified in SMM, fuzzy logic and expert judgment were used. The use of fuzzy logic in the fault tree, in addition to simplifying the evaluation, also increases the accuracy of the study (14, 29). The fault tree method is not able to analyze the dependencies between events properly. Therefore, the dependencies of some events can be found statistically using the BN model, and these CCFs can affect the final probability depending on how the CPTs are defined. For example, intermediate events Error in managing emergency Situations (B13) and Lack of risk control (B7) are statistically dependent on each other because they share a common basic event X7 (Failure to examination).

Compared with classical methods, BN provides the ability to display cause-and-effect relationships and calculate probabilities under conditions of uncertainty (29). This method has the ability to model complex patterns and nonlinear relationships between different factors, and can continuously update information based on new data (48). The application of this approach is of great importance in health systems that face high complexity and change. Events such as delays in CPR code announcement, inadequate bleeding control, and lack of team coordination had the greatest contribution to the occurrence of critical events. Prioritizing key factors can help policymakers make effective decisions. Special attention to bleeding control and improving team coordination in emergencies can reduce the likelihood of SMM.

Fast Fourier Transform and FBN (with and without CCFs) were analyzed. According to the results, the probability of morbidity error in FFT was higher than in FBN. Also, FBN with CCFs had a lower probability than the previous two cases. In this model, BN can reduce uncertainty, and examine complex causal relationships, and consecutive dependent failures. According to the studies of Meel and Seider (49), Laal et al. (14, 29), and Kalantarnia et al. (50), the use of definite AND and OR probabilities is one of the disadvantages of BN. Therefore, in this study, instead of emphasizing the use of definite AND and OR probabilities, Leaky-Noisy OR (L-NOR) gate was also used to complete CPTs in maternal morbidity. The L-NOR gate was used because different events had error or failure probabilities in addition to their existing conditional dependencies. Without that, the number of CPTs would increase exponentially with the number of parental variables (51), and the computational complexity would be very high. Therefore, this approach is recommended in similar studies.

### 4.1 Suggestions for future studies and limitations

Future research should investigate targeted interventions derived from these findings in clinical settings and evaluate their effectiveness in in reducing human error. Developing standardized rapid-response protocols and implementing continuous team-based training programs can reduce maternal complications, enhance patient safety, and improve women’s health. Also, in future studies, it would be better to use other logic and dynamic gates to analyze the results.

The use of Z-numbers is also recommended to reduce the uncertainty of expert opinions. Z-numbers allow for the representation of second-order uncertainty (both the quantity and the confidence in that quantity). Using Z-numbers in conjunction with FBN modeling would not only better represent the complexities of risk, but could also improve accurate decision-making and lead to safer strategies for mitigating SMM (52). One of the limitations of the study was the experts’ lack of familiarity with the fault tree method, which was mitigated through targeted training sessions prior to data collection; such training may be needed in future studies that include expert opinion.

## Data Availability

The original contributions presented in this study are included in this article/supplementary material, further inquiries can be directed to the corresponding author.
